# Anxiety and Depression Symptoms in COVID-19 Isolated Patients and in Their Relatives

**DOI:** 10.3389/fpsyt.2020.581598

**Published:** 2020-10-14

**Authors:** Shirel Dorman-Ilan, Nimrod Hertz-Palmor, Ayelet Brand-Gothelf, Ilanit Hasson-Ohayon, Noam Matalon, Raz Gross, Wendy Chen, Ayelet Abramovich, Arnon Afek, Amitai Ziv, Yitshak Kreiss, Itai M. Pessach, Doron Gothelf

**Affiliations:** ^1^Sheba Medical Center, Tel Hashomer, Ramat Gan, Israel; ^2^Sackler Faculty of Medicine, Tel Aviv University, Tel Aviv, Israel; ^3^Bar-Ilan University, Ramat Gan, Israel; ^4^Sagol School of Neuroscience, Tel Aviv University, Tel Aviv, Israel

**Keywords:** anxiety, depression, COVID-19, patients, relatives, children

## Abstract

**Background:** While focusing on the management and care of COVID-19 patients, the mental health of these patients and their relatives is being overlooked. The aim of the current study was to measure anxiety and depression, and to assess their association with socio-demographic and pandemic-related stress factors in COVID-19 patients and their relatives during the initial stage of hospitalization.

**Methods:** We assessed isolated hospitalized patients (*N* = 90) and their relatives (adults and children, *N* = 125) by phone, 25–72 h following patients' admission. The quantitative measures included the Anxiety and Depression modules of the Patient-Reported Outcomes Measurement Information System (PROMIS) and pandemic-related stress factors. Qualitative measures included questions exploring worries, sadness, and coping modes.

**Results:** Both patients and relatives suffer from high levels of anxiety and related pandemic worries, with lower levels of depressive symptoms. Compared to adult relatives, child relatives reported significantly lower anxiety. The multivariable logistic regression analysis revealed an increased risk for anxiety among females and a decreased risk among ultra-orthodox participants. While increased anxiety among patients was associated with feelings of isolation, increased anxiety among relatives was associated with a feeling of not being protected by the hospital.

**Conclusions:** Patients and relatives experience similar high anxiety levels which are more robust in women and lower in ultra-orthodox participants. Our findings indicate that anxiety symptoms of both patients and adult relatives should be addressed.

## Introduction

To date, empirical focus on mental health during the coronavirus pandemic (COVID-19) addressed two groups—the general population (1) and healthcare providers (2). However, evidence regarding mental health consequences of hospitalized patients with COVID-19 and their relatives is largely lacking.

Patients with COVID-19 and their relatives face a set of major stressors (3). These include social distancing from their loved ones, which increases a sense of uncertainty regarding their health status, and increased family-care burdens and economic stressors (3). The multitasking, uncertainty and strain that many patients and relatives struggle with, puts them at high risk for increased psychological distress. Yet, the mental health aspects of COVID-19 patients and their relatives are being largely overlooked (4).

The first objective of the present study, therefore, was to measure levels of anxiety and depression among COVID-19 patients and their relatives (including both adult and child relatives), during the initial stage of hospitalization. We assumed that patients and their relatives would show similar increased levels of anxiety, and that anxiety levels would be higher than depressive levels. This hypothesis is based upon the unpredictable nature of the COVID-19 and the accompanying uncertainty regarding the course of the illness and its infectious potential, which are key factors for anxiety (5). Among relatives, we hypothesized that children would show decreased anxiety and depression levels, compared to adult relatives. This hypothesis is based on the notion that although children have to deal with the same negative feeling of anxiety as adults, they do not share the same objective burdens as adults, such as caring for family function at this difficult time (6). Our second objective was to examine whether sociodemographic factors, such as sex and religiosity, and pandemic-related stress factors that have been previously identified in regards to COVID-19 and prior pandemics (1, 2), such as social isolation, would be associated with anxiety and depression levels among these populations.

## Methods

### Participants and Procedure

Between March 15th and May 1st, 2020, we approached 130 consecutive isolated patients who were hospitalized in specialized units for COVID-19 patients at Sheba Medical Center, and 158 of their first-degree relatives who lived with them prior to their hospitalization. Participants were contacted by phone, 25–72 h following their admission to the hospital, and were offered to undergo a short screening of their emotional distress. After the patient's screening, we asked for his or her consent to approach their first-degree relatives. Adult relatives underwent a similar screening procedure. Children under 18 underwent an abbreviated screening process, adjusted for a younger population. This process included questions about anxiety and depression symptoms but not about pandemic related stress.

### Measures

#### Anxiety and Depression Assessment

We used the Hebrew versions of the Anxiety and Depression modules of the Patient-Reported Outcomes Measurement Information System (PROMIS; see www.nihpromis.org) Adult and Child versions. PROMIS is a validated measure that has good agreement with more common measures such as PHQ-9 and GAD-7 (7, 8). It is used primarily for assesing a variety of mental-health domains among patients of different medical situations (9), and is suitable for adults and children older than 6 years old. PROMIS has an established mean and SD of 50 and 10, respectively. It was validated in Hebrew using standard procedure of translation and back translation by independent bilingual English-Hebrew speakers, as described previously (10, 11).

#### Pandemic-Related Stress Factors Assessment

COVID-19 related stress domains were assessed with an inventory of *pandemic-related stress factors* (PRSF). The PRSF was compiled from questions that have been shown to be pertinent in previous research on the SARS and N1H1 pandemics (12), were adjusted for the COVID-19 pandemic and applied on a population of Israeli physicians in a previous study (11). The PRSF contain questions focusing on specific worries about contagion (e.g., anxiety about infecting family), feelings of being informed and protected by the authorities, feelings of exhaustion, and social isolation. Only adults answered the PRSF items, since they were previously validated in adult populations and include contents that are not relevant for children (e.g., financial concerns, feeling protected by the government). PRSF items are presented in Table 1.

**Table 1:** Sociodemographic properties and medical evaluation of study sample.

	**Patients**	**Adult Relatives**	**Children**
Participants, *n* (%)	90 (69.2%)	91 (85.8%)	34 (65.4%)
**Dropouts**			
Unavailable, *n* (%)	24 (18.5%)	6 (5.7%)	4 (7.7%)
Language and cognitive barriers, *n* (%)	11 (8.5%)	0 (0.0%)	0 (0.0%)
Chose not to participate, *n* (%)	5 (3.8%)	9 (8.5%)	14 (26.9%)
**Characteristics**			
Age, mean **±** SD (range)^*a*^	49.3 **±** 16.0	41.9 **±** 17.0	13.0 **±** 3.2
Age, range	18–82	18–81	6–17
Sex- male/female, *n* (%)^*b*^	51/39 (56.7/43.3%)	35/56 (38.5/61.5%)	17/17 (50.0/50.0%)
Religiosity- Ultra-orthodox *n* (%)^*c*^	33 (36.3%)	31 (34.1%)	19 (55.9%)
**Familial proximity to patient**			
Spouse, *n* (%)	–	44 (48.4%)	–
Offspring, *n* (%)	–	31 (34.1%)	32 (94.1%)
Parent, *n* (%)	–	14 (15.4%)	–
Sibling, *n* (%)	–	1 (1.1%)	2 (5.9%)
**Pandemic related stress factors**	**% response often/always**		***p***
Anxiety about infecting family	36.1%	29.1%	0.34
Lack of knowledge about infectiveness and virulence	22.9%	23.1%	0.98
Lack of knowledge about protection and prevention	24.4%	17.9%	0.32
Feeling protected by the government	49.4%	39.7%	0.22
Feeling protected by the hospital	69.0%	59.7%	0.25
Financial concerns	18.8%	25.9%	0.30
Mental exhaustion	25.9%	23.7%	0.74
Sleep disorders	41.3%	27.3%	0.07
Feeling isolated and avoided by others	10.0%	9.0%	0.83

a *On independent sample t-test patients were significantly older than relatives [t_(180)_ = 3.0, p = 0.003]*.

b *On chi-square test for independence, sex distribution differed significantly between patients and relatives [$\chi_{\left(1\right)}^{2}$ = 5.6, p = 0.018]*.

c *On chi-square test for independence, differences in religiosity distribution between adult relatives and children were significant [$\chi_{\left(1\right)}^{2}$ = 4.9, p = 0.027]*.

#### Subjective Experience of Hospitalized Patients and Their Family Members

Three open-ended questions exploring worries, sadness, and coping modes were formulated to elicit spontaneous reports of participant's experiences. Specifically, the questions included: (1) “what do you worry about?”; (2) “what makes you sad?”; (3) “what assisted you to cope with worries and sad mood?.” The interviews lasted between 1 and 5 min, and were transcribed by the interviewer. Grounded theory analytic approach (13) was used to evaluate responses to the questions. Since children's answers were too short and limited in content, only adults' answers were coded.

## Statistical Analysis

### Quantitative Analysis

The PRSF were collapsed into binary values with 1 representing feeling stressed “often” or “always,” and 0 representing feeling stressed “never” or “sometimes” (11). PRSF scores were compared between groups using chi square. The PROMIS scores were coded as continuous variables (T scores) using the PROMIS coding system (14).

Within-subject differences in anxiety and depression were tested using repeated measures analysis of variance (ANOVA). We then conducted ANCOVA to compare PROMIS scores between (1) adult patients and relatives, with age, sex and religiosity serving as covariates, and (2) adult and children relatives, with sex and religiosity serving as covariates.

Linear regression models were conducted to elucidate the association between PRSF, sociodemographic properties, and mental health outcomes. Anxiety and depression were the key dependent variables, respectively, and separately for patients and their relatives. Age, sex, religiosity, and PRSF items were included as independent variables.

### Qualitative Analysis

Two raters (authors AB-G and IH-O) read the interviews, selected, and agreed upon coding themes for each category, i.e., domains of worries, domains of sadness, and coping modes. Independent coding was conducted for a subsample of 30 participants showing high inter-rater reliability with Kappa coefficients ranging from 0.92 to absolute agreement. The rest of the sample was coded by either one of the raters.

## Results

The final sample included 90 patients (69.2% of a total of 130 patients that were approached) and 125 relatives (79.1% of 158 relatives that were approached, adults, and children). The mean number of relatives per patient was 2.09 ± 1.57. 36 patients participated without their relatives, and 11 relatives participated without their hospitalized family member due to language or cognitive barriers of the patient. The rest of the participants were related to at least one other patient or family member. Sociodemographic characteristics of the study sample and the distribution of subjects excluded are presented in Table 1.

### Quantitative Results

**Figure 1:**
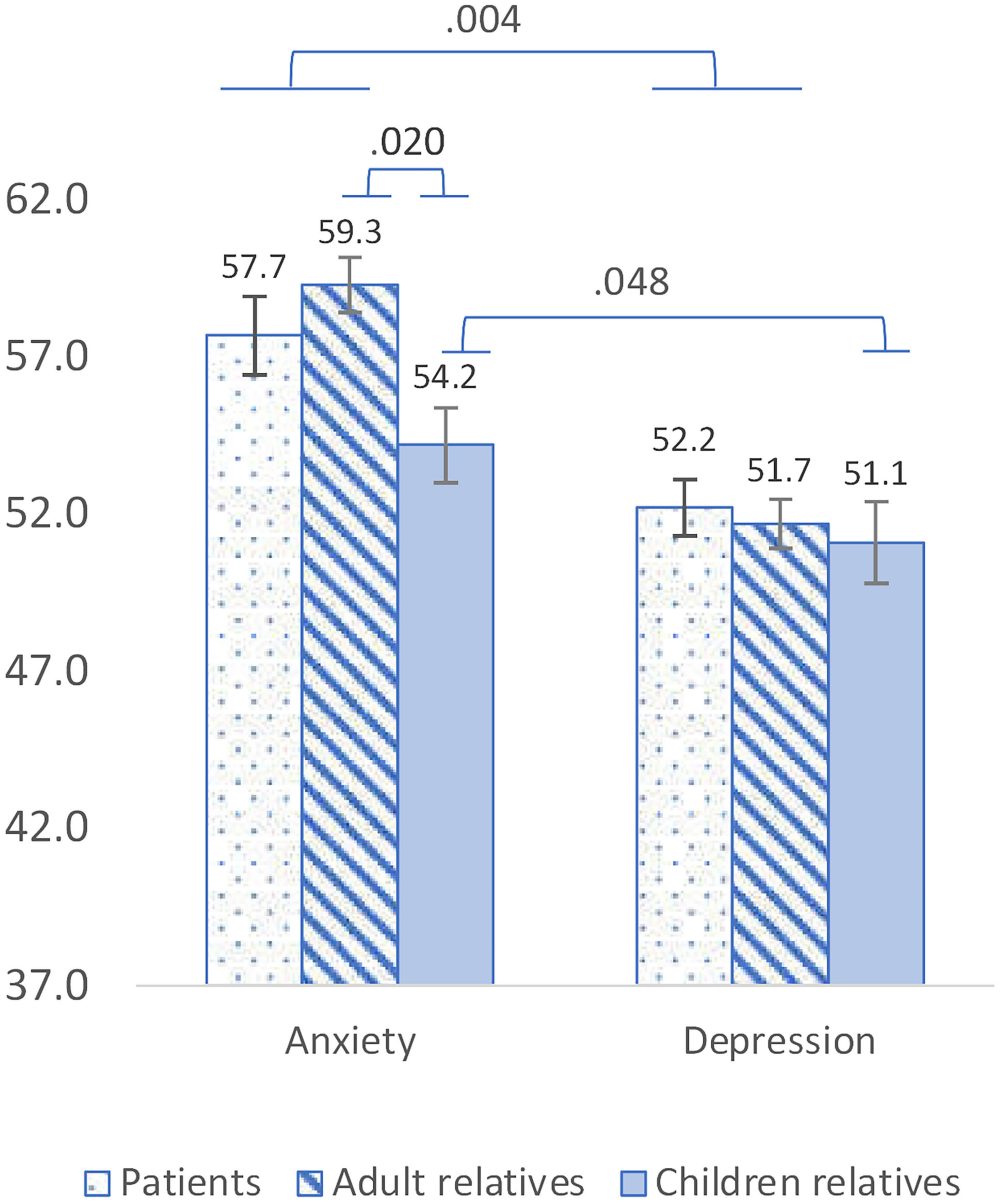
PROMIS Anxiety and Depression mean scores among adult patients, adult relatives and relatives who are children. Anxiety was significantly higher than depression among adults [*F*_(1,177)_ = 8.40, *p* = 0.004] and among children [*F*_(1,30)_ = 4.23, *p* = 0.048]. Compared to adult relatives, relatives who are children reported significantly lower levels of anxiety [*F*_(1,122)_ = 5.59, *p* = 0.02] and similar levels of depression [*F*_(1,121)_ = 0.17, *p* = 0.67].

After controlling for sex, age and religiosity, patients and adult relatives reported similar levels of anxiety [*Mean* = 57.7 ± 11.9 and 59.3 ± 8.4, respectively, *F*_(1,177)_ = 0.15, *p* = 0.69] and depression [*M* = 52.2 ± 8.5 and 51.7 ± 7.5, respectively, *F*_(1,176)_ = 0.95, *p* = 0.33] and similar degree of pandemic-related stress factors (Table 1). Anxiety was significantly higher than depression among adults, both patients and relatives [*F*_(1,177)_ = 8.40, *p* = 0.004], and among children relatives as well [*M* = 54.2 ± 7.0 vs. *M* = 51.1 ± 7.6, *F*_(1,30)_ = 4.23, *p* = 0.048]. Compared to adult relatives, relatives who are children reported significantly lower levels of anxiety [*F*_(1,122)_ = 5.59, *p* = 0.02] and similar levels of depression [*F*_(1,121)_ = 0.17, *p* = 0.67]. Anxiety and depression scores are presented in Figure 1.

In a linear regression model, sex (being a female) was associated with increased anxiety (β = 0.39, *p* < 0.0001), whilst ultra-orthodox religiosity was associated with lower anxiety (β = −0.26, *p* < 0.0001). Feeling isolated and avoided by others was associated with increased anxiety among patients (β = 0.22, *p* = 0.050), but not among their relatives. Not feeling protected by the hospital was associated with increased anxiety among relatives (β = −0.29, *p* = 0.003), but not among patients (Table 2). No factors were found to significantly effect depression among neither patients nor among relatives.

**Table 2:** Factors associated with anxiety among adult patients and their adult relatives.

	**Patients (*****n*** **=** **68)**		**Adult relatives (*****n*** **=** **66)**	
	***R*** **=** **0.596, Adj**. ***R***^****2****^ **=** **0.268**		***R*** **=** **0.726, Adj**. ***R***^****2****^ **=** **0.461**	
	**β (95% CI)**	***P***	**β (95% CI)**	***P***
Age	−0.03 (−0.26 to 0.19)	0.753	0.00 (−0.28 to 0.29)	0.975
Female	0.36 (0.14 to 0.58)	0.001	0.47 (0.28 to 0.66)	<0.0001
Religiosity—Ultra-orthodox	−0.35 (−0.59 to −0.11)	0.004	−0.18 (−0.37 to 0.00)	0.061
Social disconnection	0.22 (−0.00 to 0.45)	0.050	0.13 (−0.06 to 0.34)	0.175
Feeling protected by hospital	−0.10 (−0.34 to 0.13)	0.372	−0.29 (−0.48 to −0.10)	0.003
Anxiety about infecting family members	0.13 (−0.09 to 0.36)	0.235	0.20 (−0.03 to 0.44)	0.092
Sleep problems	0.09 (−0.14 to 0.32)	0.431	0.10 (−0.14 to 0.34)	0.399
Financial concerns	0.14 (−0.08 to 0.36)	0.211	0.11 (−0.09 to 0.31)	0.262

### Qualitative Results

The central domains of worries most commonly reported by both patients and adult relatives were uncertainty and lack of control (42.5 and 45.5%, respectively) (e.g., “I wish I could know what the prospect, how long will it last”; “he doesn't share with me his feelings; I don't know how he is doing”).

#### Domains of Worries Among Patients

32.5% of the patients reported worry about the well-being of family members at home (e.g., “I am worried about my family members both physically and emotionally, and especially about my parents who are at risk”); 25% reported worry about infecting other people, and only 17.5% reported worry about their own well-being during hospitalization (e.g., privacy, quality of food); 32.5% reported worry about their own health.

#### Domains of Worries Among Adult Relatives

73.3% of the adult relatives reported worry about their hospitalized family member; 25% worried about the mental health of patients in addition to physical health (e.g., “I'm afraid it will be too much for her emotionally”). 14.5% reported worry that their hospitalized family member will infect others.

#### Domains of Sadness

Forty-five percent of patients and 25.5% of their relatives reported that they felt sad being distant from family and friends.

#### Coping Strategies

37.5% of patients and 69.1% of their relatives agreed that support from others helped them (e.g., “feeling that people are with me, that I am not alone”). Other modes of coping were reported by <10% of the patients and relatives including religious coping, a positive approach to life, creative approach (e.g., listening to music, reading), and the use of media. Of the relatives, 27.3% mentioned that working and functioning was an important mode of coping.

## Discussion

To our knowledge, this is one of the first studies on mental health among COVID-19 patients, and the first to address relatives of confirmed patients (4). We found that both patients and relatives suffer from high levels of anxiety symptoms and related pandemic worries. While the need to routinely screen patients with medical conditions for anxiety and depression is well-established (10, 15), it was largely overlooked among COVID-19 patients and their relatives (4). There are several unique factors in COVID-19 illness that should predispose patients and relatives to heightened anxiety, including sudden deterioration of health and deaths even in young patients, uncertainty regarding length of isolation, the risk of being infected or infecting others and forced physical disconnection (3). Our qualitative interviews and responses to the PRSF indicate that indeed both patients and relatives feel uncertain and lack of control, especially with regard to length of hospitalization which depends on the results of two negative COVID-19 tests. Patients and relatives report not only worries about their own health and well-being, but also worries about the well-being of family members and fear of infecting others. These findings may suggest that care for others and being able to stay connected are major issues in patients coping with the COVID 19 and their relatives.

We also found that only among relatives, anxiety was associated with a feeling of not being protected or taken care of by the hospital. This might be explained by the fact that relatives are not allowed to visit their hospitalized relative which may negatively affect their trust in the care provided to their loved ones, which further emphasizes the importance of taking care of COVID-19 patients' relatives.

The results of our regression analyses also indicate that the risk for anxiety is increased among females and is decreased among the ultra-orthodox. The higher levels of anxiety in women compared to men are consistent with the known increased life-time rates of anxiety disorders in women in the general population (16). It could also reflect the increased burden of caring for children and households during the pandemic and the fact that women are more vulnerable than men at times of economic instability (17).

The protective effect of being ultra-orthodox on anxiety, could be explained by the notion that the orthodox society is an extreme collectivistic culture, providing social support and feelings of belonging (18), thereby potentially reducing anxiety. There is also a stigma regarding mental health in the orthodox community, as having a mental illness could hamper the match-making process (19). Thus, orthodox individuals are more reluctant to share painful experiences. Interestingly, rates of COVID-19 infection among ultra-orthodox Jews in Israel and in New York City were very high (20, 21). Taken together, lower anxiety levels may partially explain the ultra-orthodox Jews being more vulnerable to contact COVID-19, since a certain degree of anxiety is needed for taking the precautions against getting infected.

The relatively low rates of depression found in our study corresponds with a temporary decrease in the rates of suicides following national crises (22). This is explained by the “pulling together effect” whereby individuals undergoing a shared experience support one another, thus strengthening social connectedness which could mitigate depression. Based on experience with previous national crises and prior pandemics (e.g., Ebola), it is likely that a degree of depressive symptoms will increase later on (23). We found that children reported significantly lower levels of anxiety than adult relatives. Referring to the classic distinction between objective and subjective burden among family caregivers (24), the lower distress among children may be due to the fact that while both adults and children face subjective burden (i.e., sadness and fear of contamination) adults face additional objective burden (i.e., financial difficulties).

This study has several limitations. First, its cross-sectional design limits conclusions about directionality. However, the fact that all participants were screened soon after hospitalization is a strength and will enable us to conduct a follow-up of this cohort. Second, although the acceptance rate to participate in the study was high, it is possible that patients and relatives who chose not to participate were more stressed. This may result in a selection bias affecting the internal validity and generalizability of results. If exists, this bias is especially important in regards to children and patients, whom participation rates were relatively lower. Third, we had no evaluation of the patients' anxiety and depression levels before hospitalization, therefore it is possible that they were already high due to the pandemic, regardless of their hospitalization. Fourth, we did not compare COVID-19 patients to patients with other acute hospitalizations, particularly medical illnesses that include social distancing from the patients' families. Thus, we cannot tell whether the high levels of anxiety are specific to COVID-19 patients and their relatives. Nevertheless, our results highlight the need to evaluate and address the anxiety of COVID-19 patients and their relatives. Forth, even if our findings are generalizable to Israeli patients and relatives, they may not be fully applicable to other countries.

In conclusion, our data suggest that patients and relatives experience similarly high levels of anxiety which is more robust in adult women and lower in ultra-orthodox participants. Future follow-up of the same population will enable us to identify risk and protective factors for the persistent and evolution of mental health consequences in patients with COVID-19 and their relatives.

## Data Availability Statement

The data that support our findings are available on request from the corresponding author. The data are not publicly available due to privacy or ethical restrictions.

## Ethics Statement

The studies involving human participants were reviewed and approved by the study was approved by the Institutional Review Board of Sheba Medical Center, Tel Hashomer, Israel (IRS#SMC-7182-20). Written informed consent to participate in this study was provided by the participants' legal guardian/next of kin.

## Author Contributions

All authors contributed to, reviewed, and approved the final manuscript. Conceiving and designing the study: SD-I, NH-P, AB-G, IH-O, RG, WC, AAb, and DG. Data collection: SD-I, NH-P, and NM. Statistical analyses: SD-I and NH-P. Qualitative analyses: AB-G and IH-O. Data interpretation: SD-I, NH-P, AB-G, IH-O, RG, AAf, AZ, YK, IP, and DG. Writing the final manuscript: SD-I, NH-P, AB-G, IH-O, NM, and DG.

## Conflict of Interest

The authors declare that the research was conducted in the absence of any commercial or financial relationships that could be construed as a potential conflict of interest.
